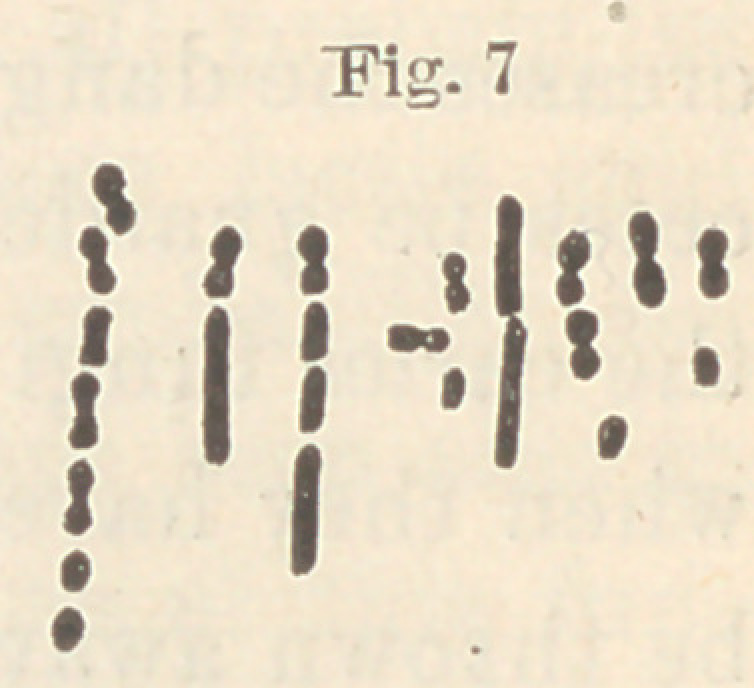# Fermentation in the Human Mouth

**Published:** 1884-05

**Authors:** W. D. Miller

**Affiliations:** Berlin, Germany


### Independent Practitioner.

VOL. V. MAY, 1884. No. 5.

### Original Communications.

### FERMENTATION IN THE HUMAN MOUTH; ITS RELATION TO CARIES OF THE TEETH.

BY DR. W. D. MILLER, BERLIN, GERMANY.

(Continued from page 119.)

If we infect a beef-extract-sugar solution with carious dentine, as described in the February number of this journal, using every possible precaution to obtain perfectly pure material and to prevent the access of germs from without, and keep the solution at thirty-seven degrees Centigrade, we may observe the following phenomena: In from eight to ten hours the solution will show a slight cloudiness, which at no time, however, amounts to complete opacity. Tested with sensitive litmus paper, it will be seen that the acid reaction has already appeared. In fifteen to twenty hours the fermentation will generally have reached the most active state, and soon afterwards a colorless, flocky precipitate will begin to form on the bottom of the vessel, accompanied by a corresponding clarifying of the solution, and a diminution of the fermentive activity. After the lapse of forty-eight hours the sediment will have completely formed, and the solution will be almost as transparent as when the experiment began.

The time required for the completion of this series of phenomena will, however, naturally depend somewhat upon the amount of dentine taken for the infection, and the amount of the solution used.

Impurities in the culture manifest themselves in various ways; it may be by an excessive cloudiness of the liquid, or by the formation of a skin upon the surface of the solution, or the failure of the latter to become clear after the regular lapse of time, etc., etc.

Dentine is an excellent medium for separating the different fungi found in the mouth, the most of them not being able to exist in the deeper parts, partly on account of the acidity of the medium, partly on account of the lack of free oxygen. We may, therefore, with the proper amount of care, obtain material of such purity as to produce a pure culture in the first generation.

If we microscopically examine the sediment which has formed on the bottom of the vessel, we shall find it to consist of cocci and diplococci, either single or in chains; in either case without motion. Under a low power they appear round and regular; with $\frac{1}{18}$ oil immersion they are seen to be round or oval, regular or irregular, involuted, etc., presenting the most various shapes and sizes. I have never been able to detect the existence of spores, and reproduction takes place only after the scheme presented in figure one, Nos. 1, 2, 3, 4, 5, 6, 7. A coccus which may be round in the beginning, by extension in one axis becomes oval or elongated; soon after it shows a contraction in the middle, resulting in the production of a diplococcus, or two cocci, each of which may produce two cocci in the same manner.

Fig. 1

We find, consequently, in a chain taken from a growing culture, some of the cocci round, others oval, some of the diplococci but slightly contracted, while in others the contraction amounts almost to a complete division. (See Fig. 1, *d, e, f.*) Frequently the cells acquire a pronounced bacterium form, so that if they did not occur in the same chain with the ordinary forms, one would be in doubt as to whether they belonged to the same species.

The growing cells in a chain sometimes turn upon their shorter axis, and then, growing out in the new direction, produce very peculiar figures. (Fig. 1, *f*, *g*.) In stagnant cultures the cells under high power are mostly very irregular, having in groups the appearance of the bones of the wrist. (See Fig. 1, *a*, *b*.)

Very characteristic are the involution forms produced both in stagnant cultures and in media which are not well adapted to the needs of the fungus. Here the forms and sizes are so various that it sometimes becomes exceedingly difficult, if not impossible, to tell if certain ones are normal or abnormal. (See Fig. 1, *h*, *i*, *j*, *k*.) In exceptional cases the threads surround themselves with a thick gelatinous sheath. (See Fig. 1, *c*.) The protoplasm of the involuted cells generally presents a granular appearance. (Fig. 1, *h*, *k*.)

If we make a large number of cultures at once we will, in about one case out of five to ten (and if the cultures are made in a decoction of malt, much more frequently), meet with a second fungus, essentially different from the one just described. It occurs chiefly in form of bacilli, but also as leptothrix, bacteria, diplococci and cocci singly, or, as is mostly the case, in long zigzag threads. (Fig. 2.)

Fig. 2

The discovery of this fungus, with its different forms of development, affords a very ready explanation of the fact that in a single dentinal tubule we sometimes find a transition from leptothrix to bacilli, from bacilli to bacteria, and from bacteria to cocci, an occurrence which I demonstrated nearly two years ago before the American Dental Society of Europe, before the Gesellschaft fuer Heilkunde, in Berlin, and to various private persons, including some of the most celebrated mycologists in Germany.

Those who maintain, as was done in the British Dental Association, that such cases may not be found, are responsible for their own mistake.

*Macro*-scopically, cultures of this fungus in beef-extract-sugar solution are not easily to be distinguished from cultures of that described above. The fungus collects as a sediment on the bottom of the vessel; it never forms a skin on the surface of the liquid, and produces but a moderate cloudiness of the same. In most decoctions, however, they present some peculiarities. Sometimes the fungus floats about in the solution in semi-transparent balls, or rises up from the bottom of the vessel like a miniature cloud of smoke, or collects in small patches on the sides of the vessel, while the solution itself remains almost perfectly clear. The cells are motionless, and do not form spores.

Fig. 3

In order to discriminate between these two fungi I will designate for the present the one first described by the prefix *A* (alpha), and the one under consideration by the prefix *β* (beta). In all probability the *β* fungus also produces lactic acid from sugar. I say in all probability, because, though I have always been able to detect lactic acid in cultures of this fungus, I could not say with absolute certainty that cocci and diplococci of the species *A* were not present.

We have, then, in carious dentine two distinct fungi—one always, the other often, present; the former surely, the latter probably, producing lactic acid from sugar. If these fungi are the direct cause of dental caries, we should be able to produce caries by subjecting sound dentine to their action. This I have accomplished, as described in the March number of this journal.

In figure three *a* are seen in outline two tubules of dentine melted together by natural caries, and in figure three *b* two tubules melted together by artificial caries.

In figure four *a* are likewise two tubules from natural caries, and in figure four *b* two from artificial caries. It is a fact of considerable interest that, though the fungi themselves are perfectly colorless, pieces of dentine subjected to their action become yellowish, light brown, or dark brown, etc., depending upon the medium in which the culture is made, while different pieces of dentine in the same culture do not by any means necessarily acquire the same color.

Fig. 4

The carrying out of this experiment is attended with difficulties, and some may try it and fail. I have failed many times. The necessity of repeatedly changing the solution very much increases the danger from impurities; especially must the saccharomycetes be guarded against. The acidity of the medium caused by the caries fungi renders it very favorable for their development, and when they have once found their way into a culture it might as well be thrown away at once. Again, notwithstanding the presence of the pieces of dentine, the solution sometimes becomes sufficiently acid to impair, if not to destroy, the vitality of the fungus. In this case the dentine becomes softened, but only a slight invasion of the tubules takes place. Then, of course, in the very last stage of caries other fungi, especially leptothrix buccalis, are present in the decomposing dentine, and sometimes produce an appearance in its *superficial* layers, which I have not attempted to reproduce artificially. It is not difficult, by a simple microscopic examination of the fluids of the mouth, as well as of carious dentine, to find forms morphologically identical with those described above.

In figure five (see next page) is seen in outline a portion of an epithelial scale from the human mouth, highly magnified, with the

Fig. 5

fungi lying upon the surface. The forms seen in figure six were obtained from a glass tube filled with starch and kept in the mouth over night, while figure seven is from carious dentine.

Fig. 6

Fig. 7

The *A* caries fungus agrees morphologically with the fungus of sour milk as delineated by Pasteur. Later experiments, however, render it probable that the souring of milk is produced by an altogether different fungus, a short, thick *bacterium*, occurring in twos, seldom fours, which may also be found in the human mouth (though probably not deep in carious dentine), and will be considered at another time.

In the case of both fungi the *fermentation* goes on independently of the presence of free oxygen. I have already shown that where only a trace of oxygen is present, in no way comparable with the amount of acid produced, the degree of acidity was as great as where there was free access of air. Whether, however, this *trace* of oxygen is essential to the life of these fungi—*i. e.*, whether without it they would perish from asphyxia—is a question which we will not discuss here.

It has been generally supposed that the production of lactic acid by fermentation from sugar is accompanied by the evolution of carbonic acid; in fact, Fluegge says that no fermentation can go on without the production of carbonic acid. This statement will hardly be borne out by a study of the fermentation produced by the fungi of tooth caries.

A glass vessel of five hundred c. c. capacity was filled with beef-extract-sugar solution, infected with a pure culture of caries fungi and made air-tight with a rubber stopper, carrying an efflux-tube for collecting the gas over mercury. After twenty-four hours, during which time 1,75 c. c. acid had been produced, one single gas-bubble was collected, which may have been due to a slight change of temperature, as well as to a veritable gas evolution. The splitting appears therefore to be perfectly smooth, and to take place in accordance with the simple formula—

$$C_6 H_{12} O_6 = 2C_3 H_6 O_3.$$

It presents a marked contrast to the stormy character of the butyric and alcoholic fermentations, in case of which the pressure of the gas evolved is often sufficient to burst the vessels containing the cultures.

There is perhaps at nearly all times a sufficient amount of sugar in the oral cavity to enable the fungi of caries to carry out their characteristic ferment action. It remains, nevertheless, an interesting question, whether they have the power to form sugar out of starch; *i. e.*, whether they have any diastatic action. About thirty cultures in an aqueous solution of beef-extract and starch, and in a solution of starch in sterilized saliva, gave for the most part negative results; in exceptional cases a slight diastatic action appeared to take place, which I am inclined to regard as the result of some impurity in the culture, or an error in the experiment.

On the other hand, the fungi appear without doubt to possess the power to invert, or to render non-fermentable sugars fermentable, since cane sugar, which is not fermentable, and does not reduce alkaline solutions of sulphate of copper, acquires both these properties when subjected to their action. That this result is caused by the action of a ferment produced by the organisms, and which may be separated from them, is, I think, demonstrated by the following experiment: By making a number of cultures at one time in vessels of two hundred to five hundred c. c. capacity, and collecting the sediment which was deposited on the bottom of the vessels, I succeeded in bringing together a considerable quantity of the fungi; this was then treated with ninety per cent. alcohol, filtered and dried in a porcelain vessel, thoroughly rubbed with sand, digested with water at twenty-three degrees Centigrade, and again filtered; the filtrate (which must be clear, and should contain the ferment in solution) was added to a solution of cane sugar, which then showed, in the long tube of a Mitscherlich polariscope, a rotation equal to 5°,19. The solution was now allowed to stand four hours at a temperature of thirty-eight degrees Centigrade, after which time it produced a rotation of only 4°,54, indicating a decrease of about two-thirds of a degree. The solution also produced a slight reduction of an alkaline solution of sulphate of copper; *i. e.*, a certain portion of the cane sugar had been converted into invert sugar.

In the presence of the fungi the non-fermentable sugar, by the action of the invertine produced by the fungi, takes up one molecule of water and is coverted into invert sugar, a mixture of levulose and dextrose, both of which are fermentable.

$$C_{12} H_{22} O_{11} + H_2 O = C_6 H_{12} O_6 + C_6 H_{12} O_6.$$

Cane sugar, Levulose, Dextrose.

We may say, therefore, that the micro-organisms require sugar to produce fermentation, but that it is immaterial which kind of sugar is furnished them. The fermentation is most active between the temperatures thirty-five and forty degrees Centigrade. Above fifty degrees and below fifteen degrees Centigrade, little or no production of acid takes place.

In addition to these two species of fungi, others of minor importance are occasionally met with in the mouth, and will receive attention at another time.

I would not have any one think that I look upon the above as a thorough consideration of the fungi of tooth caries. To me it appears very imperfect. Nevertheless, I thought it well to present the matter before the profession in the hope that others might be induced to take it up and help to complete the work thus begun. In the next number I will present the results of experiments relating to the action of various antiseptics, filling materials, etc., upon the fungi under consideration.

## Figures and Tables

**Fig. 1 f1:**
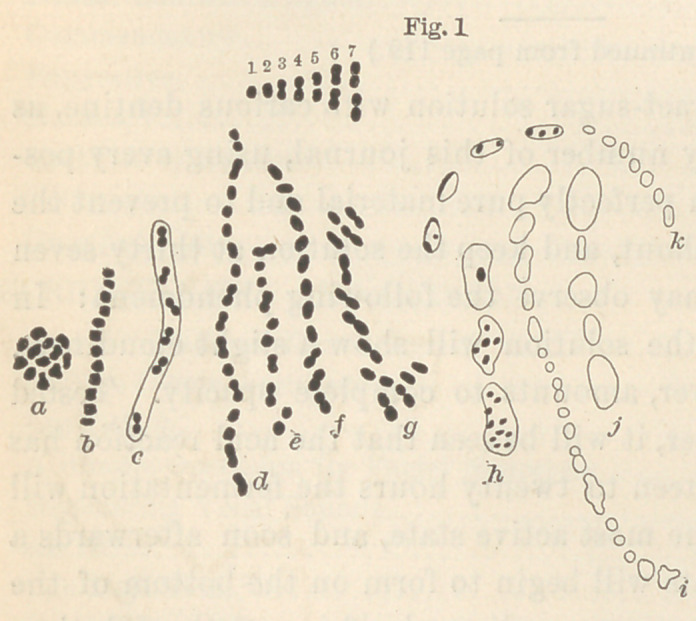


**Fig. 2 f2:**
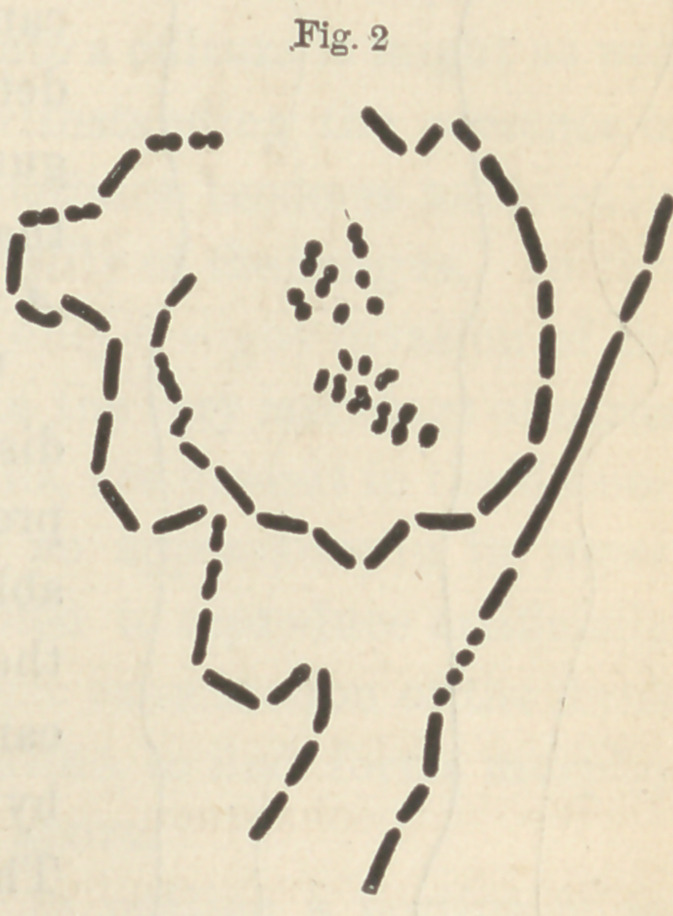


**Fig. 3 f3:**
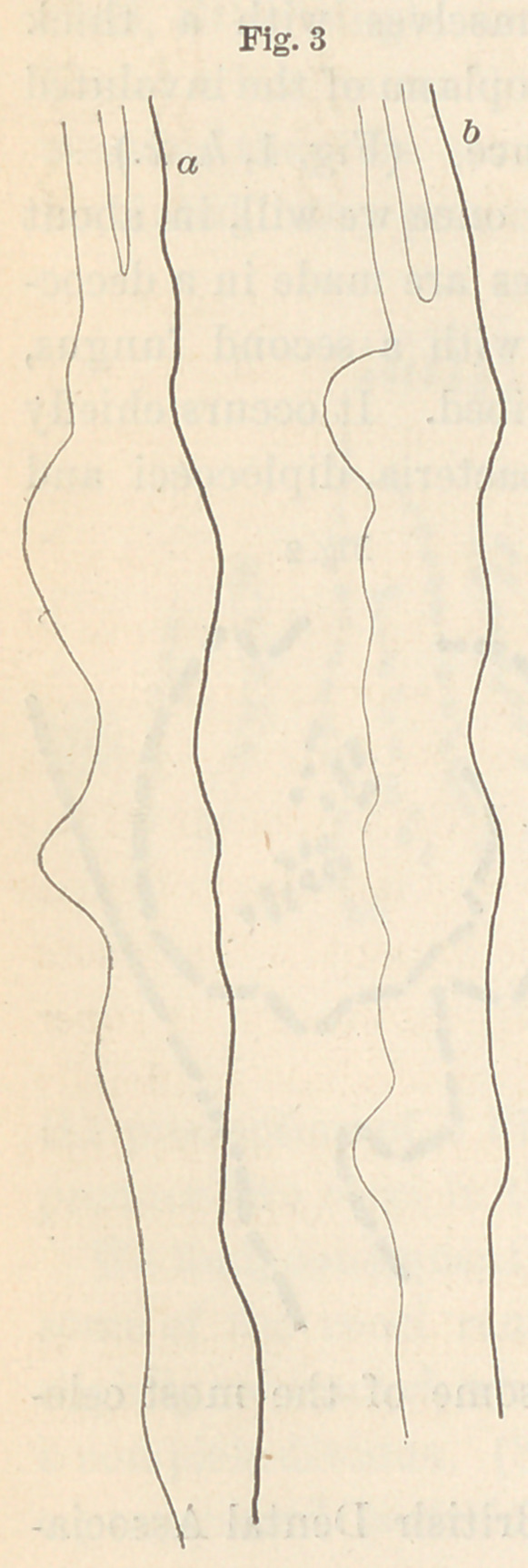


**Fig. 4 f4:**
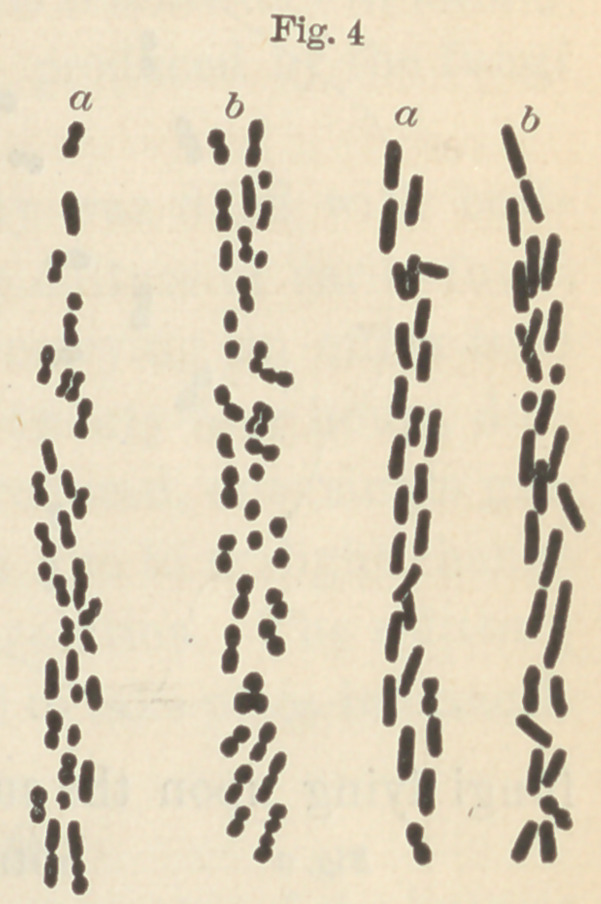


**Fig. 5 f5:**
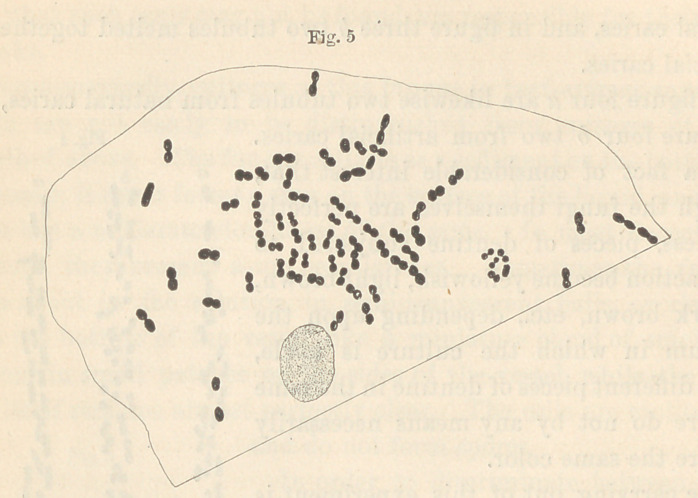


**Fig. 6 f6:**
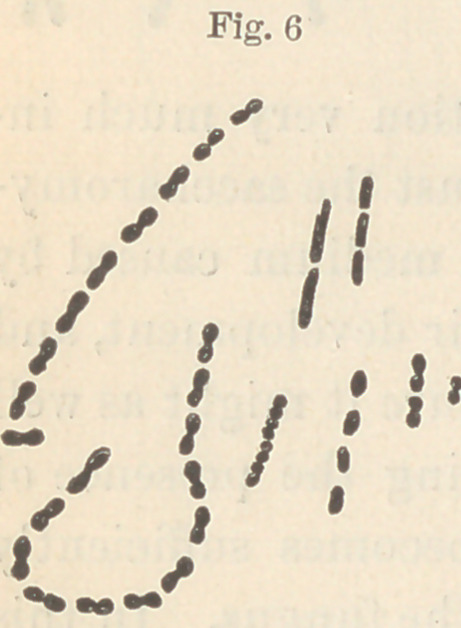


**Fig. 7 f7:**